# Identification of a selective glucocorticoid receptor ligand for the treatment of chronic inflammation in type 2 diabetes mellitus

**DOI:** 10.3892/etm.2014.1860

**Published:** 2014-07-23

**Authors:** HAIFENG TAN, WEI WANG, XIANGANG YIN, YAO LI, RUI YIN

**Affiliations:** 1 Health Examination Center, The Second People’s Hospital of Jinan, Shandong 250001, P.R. China; 2 Community Health Service, The Second People’s Hospital of Jinan, Shandong 250001, P.R. China; 3 The Cardiovascular Center, Shandong Provincial Hospital Affiliated to Shandong University, Jinan, Shandong 250021, P.R. China; 4 Department of Comprehensive Interventional Therapy, General Hospital of Jinan Military Area Command, Jinan, Shandong 250021, P.R. China; 5 Department of Reproductive Medicine, Reproductive Hospital Affiliated to Shandong University, Jinan, Shangdong 250021, P.R. China

**Keywords:** glucocorticoid receptor, selective ligand, anti-inflammatory therapy

## Abstract

The present study aimed to identify a new selective glucocorticoid receptor (GR) ligand for the treatment of chronic inflammation in type 2 diabetes mellitus. The IN Cell Analyzer 1000 platform was employed to screen for compounds that may promote GR nuclear translocation. A mammalian two-hybrid system and transactivation assay-were used to analyze the selected GR ligands and evaluate their activities for GR transcription and the recruitment of co-activators. A novel selective GR ligand, compound Q40, was identified that was able to promote GR nuclear translocation in a short period of time. It increased the ability of GR to recruit co-activators in a concentration-dependent manner, but had no positive effect on GR transcriptional activity. In conclusion, an increase in the expression levels of gluconeogeneic genes, induced by the transcriptional activation of GR, is the predisposing factor most commonly associated with the side-effects of glucocorticoids. The results suggest that compound Q40 is a ligand of the GR and exerts an agonistic action on the recruitment of co-activators without sugar dysmetabolism-related side-effects. Thus, compound Q40 has the potential to be used as an anti-inflammatory adjuvant therapy with minimal side-effects in patients with type 2 diabetes mellitus.

## Introduction

Type 2 diabetes mellitus is a glycolipid metabolic disorder that is characterized by high levels of blood glucose and lipids in the context of insulin resistance and relative insulin deficiency. It is a clinical syndrome caused by a combination of environmental and genetic factors. When insulin resistance occurs, the insulin signaling pathways of cells have a reduced ability to respond to the action of the hormone insulin. To compensate for the insulin resistance, the pancreas secretes greater amounts of insulin to stimulate glucose uptake in the surrounding tissues. A previous study has revealed that the abnormal accumulation of lipids and the increase in inflammatory factors, including tumor necrosis factor (TNF) α and interleukin (IL)6, play an important role in the pathogenesis of insulin resistance (1).

As a member of the nuclear receptor superfamily, the glucocorticoid receptor (GR) is a ligand-activated transcription factor that regulates the associated gene expression of glucocorticoids (2). In the absence of glucocorticoids, the GR remains in the cytosol in the form of complexes with a variety of proteins, including heat shock protein 90 (Hsp90). Once glucocorticoids diffuse through the cell membrane into the cytoplasm and bind to the glucocorticoid receptor (GR), the heat shock proteins are released. The resulting activated form of GR has two principal mechanisms of action: nongenomic and genomic, as described below. It has been demonstrated that the GR influences the key molecules of certain signaling pathways through rapid nongenomic effects, including the phosphoinositide 3-kinase (PI3K), c-Jun N-terminal kinase (JNK), 14-3-3 protein and T cell receptor (TCR) signaling pathways, which are able to regulate the expression of certain pro-inflammatory factors (3). The GR also acts as a transcription factor to activate downstream gene expression in the nucleus via two mechanisms (4). One direct mechanism of action involves homodimerization of the GR, its translocation through active transport into the nucleus and binding to glucocorticoid response elements which are located in the promoter regions of certain genes. It subsequently recruits transcription factors or co-activators, changes the structure of the chromosome and regulates the expression of genes associated with sugar dysmetabolism (5). The other mechanism of GR action occurs without dimerization or combination with DNA. Activated GR complexes, with other transcription factors attached, prevent these transcription factors from binding to their target genes and thus repress the expression of inflammatory genes (6).

The regulatory effect of the GR on inflammatory genes has led to it becoming the main target for the development of anti-inflammatory agents (7). Currently, first-line anti-inflammatory agents, including dexamethasone (Dex), exert anti-inflammatory effects by activating GRs (8). As a complete agonist of GR, Dex fully facilitates the entry of GRs into the nucleus. While inhibition of the inflammation by Dex is efficient, the clinical application of Dex is limited due to severe side-effects, including central obesity (9).

The side-effects of Dex are a result of the increased expression of gluconeogenic genes, which is caused by the transcriptional activation of GR, while the anti-inflammatory action results from the transrepression of GR (10). In the view of previous studies, the development of a selective GR ligand with few side-effects has become a new development direction and research strategy for the treatment of chronic inflammation, which may be applied to the remission of type 2 diabetes mellitus (11). Thus, the aim of the present study was to develop a screening system for selective GR modulators and carry out the screening and evaluation of a sample compound library. The discovery of novel selective glucocorticoid receptor ligands may lay a foundation for the further development of therapeutic agents for chronic inflammation.

## Materials and methods

### Cell culture

Cultures of 293T (American Type Culture Collection, Manassas, VA, USA) and human osteosarcoma (Thermo Fisher Scientific, Inc., Rockford, IL, USA) cell lines were stably transfected with GR-green fluorescent protein (GFP) in a carbon dioxide incubator at 37°C in 5% CO_2_. The Dulbecco’s modified Eagle’s medium (DMEM) containing 10% fetal bovine serum (FBS; Gibco^®^, Invitrogen Life Technologies, Grand Island, NY, USA) and 10% penicillin-streptomycin (Gibco^®^, Invitrogen Life Technologies) was changed every day. Pancreatic enzyme (Gibco^®^, Invitrogen Life Technologies) was applied every two days, and the cells were cultured for 14 days in total. Following the subculture, cells in the logarithmic growth phase were seeded in 96-well plates and cultured in the incubator overnight.

### Screening of compounds

The compounds in the compound sample library [preserved in the laboratory at the Second People’s Hopital of Jinan (Jinan, China)] were dissolved in serum-free DMEM to incubate with the cells stably transfected with GR-GFP for 1 or 6 h. Following the removal of DMEM, cells were treated with a fixative containing Hoechst 33342 (Beyotime Institute of Biotechnology, Haimen, China), at a final concentration of 1 mg/ml, and methanal, at a final concentration of 4%, for 30 min. Cells were washed three times with phosphate-buffered saline (PBS) and analyzed using the IN Cell Analyzer 1000 (GE Healthcare, Bethesda, MD, USA). Dimethyl sulfoxide (DMSO) served as the negative control and Dex (Sigma, St. Louis, MO, USA) served as the positive control.

### Luciferase assay

A luciferase assay was performed to determine the effect of compound Q40 on GR transcriptional activity and its ability to recruit co-activators. The 293T cells in the logarithmic growth phase were seeded onto 24-well plates and cultured in the carbon dioxide incubator. When cell density reached 50–70%, the medium was changed to serum-free DMEM. A Calcium Phosphate Transfection kit (Beyotime Institute of Biotechnology) was used to transfer relevant plasmids into the cells. The GR-ligand-binding domain (LBD), UAS-TK-Luc reporter and pRL-SV40 control plasmid were transferred into the 293T cells during the investigation of the ability of GR to recruit co-activators. GR full-length plasmids, the glucocorticoid response element-luciferase (GRE-Luc) and pRL-SV40 control plasmid were transferred into the 293T cells during the detection of the influence of compounds on GR transcriptional activity. The culture medium was replaced with complete medium with 10% FBS following a 6-h transfection. The compounds in the compound sample library were dissolved in the complete medium and the cells were incubated in the medium for 18 h. Following the removal of DMEM, cells were washed once with PBS. A total of 100 μl cell lysis solution was added to each well to lyse the cells at 37°C. Cells were harvested within the 20 min subsequent to lysis. A luciferase assay was performed according to the manufacturers’ instructions (Promega Corporation, Madison, Wisconsin, USA).

### Statistical analysis

SPSS software, version 16.0 (SPSS, Inc, Chicago, IL, USA) was used to carry out the statistical analysis. Data were compared using the Student’s t-test. P<0.05 was considered to indicate a statistically significant difference.

## Results

### Compound Q40 significantly promotes GR nuclear translocation

**Figure 1:**
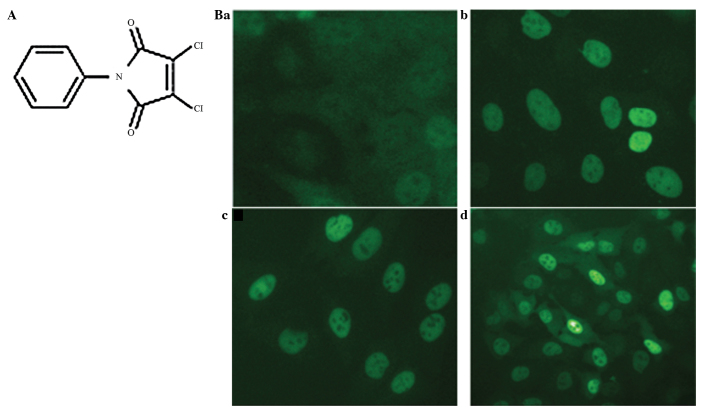
(A) Structural formula of compound Q40. (B) Compound Q40 significantly promoted the entry of glucocorticoid receptor-green fluorescent protein (GR-GFP) into the nucleus. (a) Dimethy sulfoxide (negative control). (b) Dexamethasone (positive control). (c and d) Compound Q40 promoted the entry of GR-GFP into the nucleus at (c) 6 and (d) 1 h, and the effect of compound Q40 on GR-GFP nuclear translocation was significant. Magnification, ×400.

Following screening of the compounds in the compound sample library, the compound Q40 was identified to significantly promote GR nuclear translocation. A total of 20 μm Q40 was able to promote the entry of GR-GFP into the nucleus in the shortest time of 1 h (Fig. 1). DMSO served as the negative control and Dex as the positive control. As the regulating effect of GR is mainly exerted in the nucleus, a mammalian two-hybrid system and transactivation assay were used to investigate the effect of compound Q40 on the GR.

### Compound Q40 is a selective regulator of the GR

**Figure 2:**
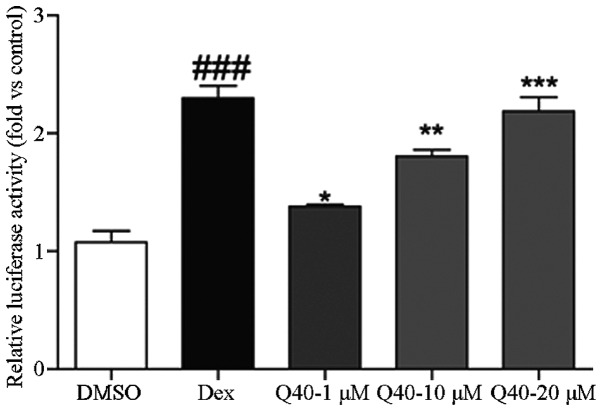
Compound Q40 increased the ability of glucocorticoid receptor (GR) to recruit co-activators in a concentration-dependent manner. DMSO, dimethyl sulfoxide (negative control); Dex, dexamethasone (positive control). ^###^P<0.001; ^*^P<0.05; ^**^P<0.01; ^***^P<0.001 vs. DMSO.

A mammalian two-hybrid system was used to investigate the effect of compound Q40 on the ability of GRs to recruit co-activators. At 6 h following transfection, the 293T cells were treated with different concentrations of compound Q40 (1, 10 and 20 μmol/l), DMSO or Dex for 18 h. The luciferase assay revealed that compound Q40 increased the ability of GRs to recruit co-activators in a concentration-dependent manner (Fig. 2).

### Compound Q40 has no effect on GR transcriptional activity

**Figure 3:**
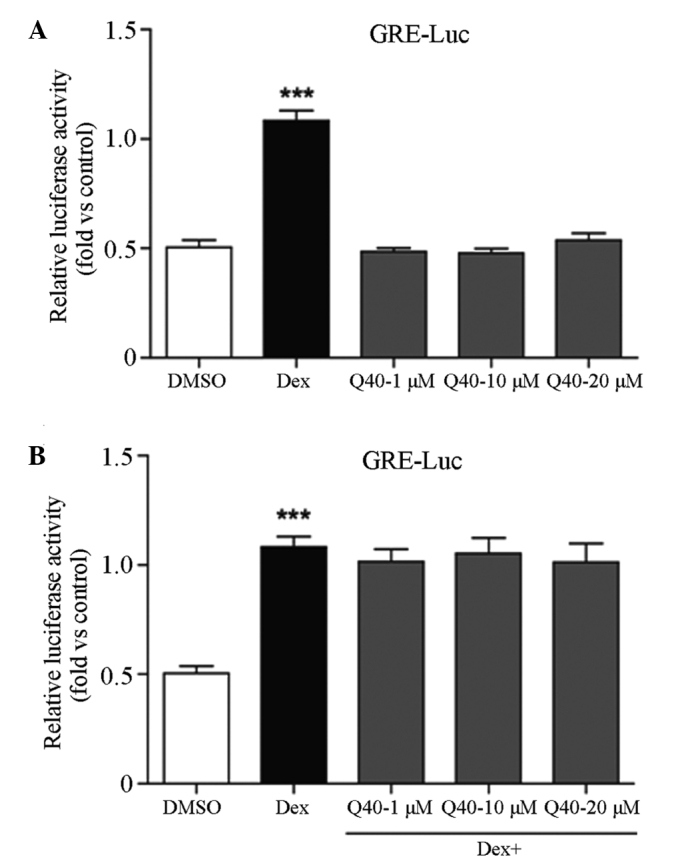
(A) Compound Q40 had no effect on the transcriptional activity of the glucocorticoid receptor (GR). (B) Compound Q40 had no effect on the partial agonistic action of dexamethasone (Dex) on the glucocorticoid response element (GRE). The negative control was dimethyl sulfoxide (DMSO) and the positive control was Dex. Luc, luciferase.^***^P<0.001 vs. DMSO.

At 6 h following transfection, 293T cells were treated with various concentrations of compound Q40 (1, 10 and 20 μmol/l), DMSO or Dex for 18 h. The luciferase assay revealed that compound Q40 had no effect on the transcriptional activity of GR. When the 293T cells were incubated with Q40 and Dex combined, compound Q40 had no effect on the partial agonistic action of Dex on GRE. This indicates that Q40 is not an antagonist of the GR (Fig. 3).

## Discussion

Type 2 diabetes mellitus has become a burden on economic and social development due to its high morbidity rate. It is a metabolic disorder in the context of insulin resistance and relative insulin deficiency. Chronic inflammation plays a major role in the occurrence of insulin resistance. The transcription and expression of inflammatory factors, including TNF-α and IL-6, are activated in patients with type 2 diabetes mellitus. Increasing the sensitivity of insulin signaling through anti-inflammatory therapy has become an important strategy for the development of therapeutic agents for individuals with type 2 diabetes mellitus (12).

As a member of the nuclear receptor superfamily, the GR is typical of the ligand-activated transcription factors. Upon binding to its respective ligands, including glucocorticoids and steroid hormones, the GR is activated. The activated GR is transported into the nucleus to regulate the expression levels of inflammatory and sugar dysmetabolism-related genes, including nuclear factor κ-light-chain-enhancer of activated B cells (NF-κB), TNF-α, glucose 6-phosphatase (G6Pase) and phosphoenolpyruvate carboxykinase (PEPCK) (13). Its ability to regulate inflammatory gene expression has led to the GR becoming the primary target for the development of anti-inflammatory agents (14). As a typical first-line anti-inflammatory agent, the clinical application of Dex is limited due to severe side-effects, which are induced by the transcriptional activation of GR (15,16). The increased expression of gluconeogeneic genes, including G6Pase and PEPCK, is the predisposing factor most commonly associated with the side-effects of glucocorticoids (17,18). Therefore, the screening of selective ligands of the GR is an important strategy for the development of anti-inflammatory agents with few side-effects.

In the present study, the IN Cell Analyzer 1000 platform was employed to select compounds that were able to promote GR nuclear translocation. Following screening of all compounds in the compound sample library, compound Q40 was identified to accelerate the translocation of GR into the nucleus in a short time. A mammalian two-hybrid system and transactivation assay were performed to evaluate the regulatory effect of compound Q40 on GR function. The results revealed that Q40 increased the ability of GR to recruit co-activators in a concentration-dependent manner but had no effect on the transcriptional activity of GR itself. In light of these results, it is evident that compound Q40 is a selective regulator of GR.

In the current study, compound Q40 demonstrated a promotional effect on GR nuclear translocation and the ability of GR to recruit co-activators. As the effect of GRs on the regulation of inflammatory gene expression is carried out in the nucleus, the ability of Q40 to translocate GRs to the nucleus indicates that it may regulate the expression of inflammatory genes.

Since compound Q40 did not increase or have an antagonistic effect on the transcriptional activity of GR, the results indicated that compound Q40 did not enhance the regulatory effect of the GR on the expression of genes associated with sugar dysmetabolism. Thus, the results indicated that compound Q40 may not cause sugar dysmetabolism-related side-effects. However, identification of the biological function of compound Q40 requires further study.

As a nuclear receptor, the GR is the main target for first-line anti-inflammatory agents. Chronic inflammation plays a major role in the occurrence of insulin resistance. Therefore, screening selective ligands of the GR is an important strategy for the further development of anti-inflammatory adjuvant therapy. The results of the present study suggest that compound Q40 is a ligand of GRs and exerts an agonistic action on GRs in the recruitment of co-activators in a concentration-dependent manner, without sugar dysmetabolism-related side-effects. Thus, compound Q40 may be used as an anti-inflammatory adjuvant therapy with few side-effects in patients with type 2 diabetes mellitus.
